# Homeopathy and acupuncture teaching at Faculdade de Medicina da Universidade de São Paulo: the undergraduates’ attitudes

**DOI:** 10.1590/S1516-31802005000200009

**Published:** 2005-03-02

**Authors:** Marcus Zulian Teixeira, Chin An Lin, Milton de Arruda Martins

**Keywords:** Medical education, Complementary therapies, Homeopathy, Acupuncture, Attitude, Educação médica, Medicina complementar, Homeopatia, Acupuntura, Atitude

## Abstract

**CONTEXT AND OBJECTIVE:**

Homeopathy and acupuncture, although recognized as medical specializations in Brazil, are not taught in most medical schools. The objective was to evaluate undergraduate attitudes towards them following their inclusion as optional disciplines at Facul-dade de Medicina da Universidade de São Paulo (FMUSP) in 2002.

**DESIGN AND SETTING:**

Questionnaire, at FMUSP.

**METHODS:**

484 students answered a self-administered questionnaire on these therapies, regarding their interest in learning, the teaching methods, their knowledge/experience (or that of someone close to them) and how it was acquired, the main indicators and general effectiveness of these therapies, and the possibilities for offering and integrating them within public healthcare units.

**RESULTS:**

Over 85% of the students considered that homeopathy and acupuncture should be included in curricula, as options (72%) or compulsorily (19%); 56% showed great interest in learning about them. Although 76% had little or no knowledge, 67% believed that these therapies had some effectiveness, and that chronic diseases (37%) or even chronic and acute diseases (29%) would be the main indicators for their use. Around 35% were receptive towards offering public primary care using both therapies, while 34% thought these treatments should also be available in hospitals and 60% believed they could be integrated with conventional medical practices.

**CONCLUSION:**

The medical students were interested in learning the principles of homeopathy and acupuncture, were able to observe and report on the effectiveness of these treatments and defended the use of these medical specializations within public healthcare.

## RESUMO

**CONTEXTO E OBJETIVO:**

Apesar de a homeopatia e a acupuntura serem reconhecidas como especialidades médicas no Brasil, não são ensinadas na maioria das escolas médicas. O objetivo foi avaliar as atitudes dos acadêmicos perante estas práticas, após sua inserção como disciplinas eletivas no currículo da Faculdade de Medicina da Universidade de São Paulo (FMUSP) em 2002.

**TIPO DE ESTUDO E LOCAL:**

Questionário, na FMUSP.

**MÉTODOS:**

484 estudantes responderam um questionário auto-aplicável sobre interesse no aprendizado, forma de ensino, nível de conhecimento e forma de aquisição, experiência da terapêutica em si próprios ou pessoas próximas, principais indicações e eficácia geral, e possibilidade de oferecimento e integração junto aos serviços públicos de saúde.

**RESULTADOS:**

Acima de 85% dos estudantes considerou que a homeopatia e a acupuntura deveriam estar inseridas no currículo, de forma opcional (72%) ou obrigatória (19%); 56% mostraram-se muito interessados no aprendizado. Apesar de 76% terem nenhum ou pouco conhecimento, 67% creditavam algum grau de eficácia às terapêuticas, tendo como principais indicações as doenças crônicas, isoladamente (37%) ou englobando também as doenças agudas (29%). Em torno de 35% foram favoráveis ao oferecimento ambulatorial nos serviços públicos de saúde, enquanto que 34% defenderam a disponibilização também em hospitais, com 60% acreditando na possibilidade de integração com a prática médica convencional.

**CONCLUSÕES:**

A maioria dos estudantes de medicina entrevistados mostrou-se bastante interessada em aprender os fundamentos da homeopatia e da acupuntura durante a graduação, foi capaz de observar e reportar a eficácia e defendeu a incorporação nos serviços públicos de saúde.

## INTRODUCTION

Popular interest in complementary and alternative medicine (CAM) in Brazil is as large as it is in other countries,^1–4^ especially with regard to phytotherapy, homeopathy and acupuncture.

In view of the growing interest in learning about CAM among medical professionals^5–8^ and medical students, medical schools in several countries have, over the last few years, started to include the teaching of these therapeutic methods in the basic undergraduate curriculum.^9^

A survey in the United Kingdom in 1996^10^ showed that 23% of the medical schools had incorporated into their curricula some disciplines that provided basic concepts regarding the various forms of CAM. In 1999, 40% of medical schools in the European Union were offering CAM courses.^11^ In 1997-1998, a survey of 117 American medical schools^12^ showed that 64% of them had CAM courses; in a more recent survey^13^ this figure increased. A survey among Canadian medical schools in 1998 indicated that 81% of them presented CAM topics in their curricula.^14^ A survey in 80 Japanese medical schools^15^ in 1998-1999 showed that 20% of them taught CAM, in a total of 25 courses, especially the teaching of acupuncture.

There are few medical schools in Brazil that include the systematic teaching of homeopathy and acupuncture as compulsory or optional (elective) disciplines within their curricula. The medical profession is thus deprived of guidance regarding the basic principles and scientific evidence that support such therapy. These are indispensable tools for providing appropriate guidance to patients regarding the use of these medical practices, as well as on the risks involved.^16–18^

In a repetition of the initiative undertaken in other countries for estimating medical undergraduates’ interest in and knowledge of CAM practices, we therefore conducted a survey on the attitudes of the students of Faculdade de Medicina da Universidade de São Paulo (FMUSP) concerning homeopathy and acupuncture, and also on their attitudes towards the initiative of starting to teach these medical specializations at this university.

In 2002, these subjects were introduced into FMUSP's undergraduate curriculum as elective disciplines, with the objective of satisfying undergraduates’ interest in learning the principles, scientific evidence and clinical practice of these branches of medicine.

With the aim of improving this initiative, we drew up a self-administered questionnaire for evaluating the attitudes among FMUSP students regarding learning about homeopathy and acupuncture, in order to obtain a profile that may contribute to the organizing of learning activities relating to these recently implemented subjects in FMUSP's medicine course.

## METHODS

The survey included undergraduates from all six years of the undergraduate medical course. The self-administered questionnaire was presented to the students at the beginning or end of a single teaching activity (class or test), and they were asked to fill out the questionnaire immediately after that activity.

The total number of students consulted was 512, of whom 484 returned their questionnaires correctly filled out (94.5%). The breakdown by undergraduate year was as follows: 111 (1^st^ year), 85 (2^nd^ year), 73 (3^rd^ year), 64 (4^th^ year), 85 (5^th^ year) and 66 (6^th^ year).

## RESULTS

Overall, 59.1% of the participants were men and 40.9% were women. Regarding their future medical specialization, 44.9% of the students interviewed had not made any choice by that time, while 55.1% of them mentioned one or more options.

Most of the students had a favorable attitude towards including the teaching of the principles of homeopathy and acupuncture in medical school curricula, either as optional disciplines (homeopathy: 70.6%; acupuncture: 73.7%) or compulsory subjects (homeopathy: 15.1%; acupuncture: 22.3%). The following arguments were used in justification of their opinions: "the physician needs to know every specialization"; "the physician should at least be able to discuss these treatments or recommend them to patients, because there is a large demand for them"; and "before criticizing them (due to prejudice), the physician needs to know the principles". Some undergraduates who did not support the teaching of these specializations justified their position by arguing that these specializations "do not present a scientific basis" (Table 1).

**Table 1:** Students’ attitudes towards including homeopathy and acupuncture in the undergraduate curriculum and their interest in learning about these subjects, at Faculdade de Medicina da Universidade de São Paulo (n = 484)

Undergraduate years	Are you in favor of including homeopathy and acupuncture in the undergraduate curriculum? (%)								Are you interested in learning about homeopathy and acupuncture? (%)					
	No		As an option		Compulsorily		Don't know		Not interested		A little		A lot	
	Hom	Acu	Hom	Acu	Hom	Acu	Hom	Acu	Hom	Acu	Hom	Acu	Hom	Acu
1^st^ year	2.7	0.9	72.1	76.6	21.6	20.7	3.6	1.8	7.2	0.9	33.3	18.9	59.4	80.2
2^nd^ year	8.2	1.2	72.9	80.0	14.1	17.6	4.7	1.2	9.4	1.2	43.5	30.6	47.0	68.2
3^rd^ year	6.8	4.1	75.3	75.3	15.1	20.5	2.7	0	9.6	2.7	35.6	39.7	54.8	57.5
4^th^ year	10.9	1.6	70.3	78.1	14.1	18.7	4.7	1.6	14.1	3.1	46.9	32.8	39.1	64.1
5^th^ year	18.8	4.7	68.2	62.3	10.6	31.8	2.3	1.2	16.5	2.3	45.9	28.2	37.6	69.4
6th year	19.7	6.1	65.1	69.7	15.1	24.2	0	0	27.3	7.6	43.9	30.3	28.8	62.1
**% Total**	**11.2**	**3.1**	**70.6**	**73.7**	**15.1**	**22.3**	**3.0**	**1.0**	**14.0**	**3.0**	**41.5**	**30.1**	**44.4**	**66.9**

*Hom = Homeopathy; Acu = Acupuncture.*

The vast majority of these students showed some interest in getting to know about these therapeutic proposals (homeopathy: 85.9%; acupuncture: 97.0%). A significant proportion demonstrated reasonable interest in the learning about them (homeopathy: 45.4%; acupuncture: 66.9%), stating that this attitude "would certainly add value to the medical course" and that "people cannot criticize or praise something without knowing about it". Among the women, 62.2% of them had "a lot of" interest in learning about homeopathy and acupuncture, while 53.8% of the men said this (Table 1).

The level of the students’ knowledge of these specializations was very low: 83.7% of them had "little or no" knowledge of homeopathy and 69.0% had "little or no" knowledge of acupuncture. It was also found that 16.3% of the students had "some or a lot of" knowledge of homeopathy, and 30.9% had "some or a lot of" knowledge of acupuncture. Among the students who said they had some knowledge, it had mainly been acquired via supplementary reading (homeopathy: 49.1%; acupuncture: 42.6%), and also via extracurricular courses (homeopathy: 8.4%; acupuncture: 21.3%) and curricular activities (homeopathy: 0.9%; acupuncture: 6.8%). Around 5.5% of the students mentioned that they acquired the knowledge "by hearsay, from people who had undergone such treatment", "by listening to the opinions of other physicians or teachers", "by talking to practicing physicians" and "by watching TV documentaries". No response to this question was given by 8.7% in relation to homeopathy and 9.1% in relation to acupuncture (Table 2).

**Table 2:** Level of knowledge of homeopathy and acupuncture, and manner in which it had been acquired, among medical students at Faculdade de Medicina da Universidade de São Paulo (n = 484)

Undergraduate years	Level of students' knowledge (%)								Manner of acquiring this knowledge (%)							
	No knowledge		Little		Some		A lot		Curriculum		Extracurricular study		Supplementary reading		Others	
	Hom	Acu	Hom	Acu	Hom	Acu	Hom	Acu	Hom	Acu	Hom	Acu	Hom	Acu	Hom	Acu
1^st^ year	17.1	8.1	63.1	58.5	16.2	28.8	3.6	4.5	2.7	17.1	2.7	4.5	60.4	54.9	4.5	6.3
2rd year	25.9	18.8	64.7	64.7	7.0	14.1	2.3	2.3	0	2.3	4.7	12.9	58.8	51.8	3.5	3.5
3^rd^ year	31.5	16.4	46.6	52.0	17.8	24.6	4.1	6.8	0	5.5	10.9	21.9	45.2	41.1	4.1	8.2
4^th^ year	34.4	18.7	53.1	56.2	10.9	21.9	1.6	3.1	1.6	7.8	12.5	25.0	29.7	26.6	7.8	7.8
5^th^ year	27.0	9.4	54.1	51.8	16.5	34.1	2.3	4.7	1.2	3.5	15.3	37.6	45.9	37.6	2.3	2.3
6th year	33.3	10.6	51.5	48.5	10.6	36.4	4.5	4.5	0	4.5	4.5	25.7	54.5	43.9	6.1	10.6
**% Total**	**28.2**	**13.7**	**55.5**	**55.3**	**13.2**	**26.6**	**3.1**	**4.3**	**0.9**	**6.8**	**8.4**	**21.3**	**49.1**	**42.6**	**4.7**	**6.4**

*Hom = Homeopathy; Acu = Acupuncture.*

Around 82% of the students reported either that they had undergone homeopathy or acupuncture treatment themselves or that someone they knew well had done so. Among these students, some said the result had been ineffective (homeopathy: 17.7%; acupuncture: 4.1%) but, on the other hand, 56.8% (home-opathy) and 78.2% (acupuncture) noticed some level of effectiveness. Twenty-one percent of the students who had had some therapeutic experience did not know how to define the result from the treatment. Some of them said that they did not remember, "because the treatment was carried out during their childhood", and the others believed that "the cure would come spontaneously" (Table 3).

**Table 3:** Medical students’ experience (personal or from someone they know well) regarding homeopathy or acupuncture treatment and results (Faculdade de Medicina da Universidade de São Paulo; n = 484)

Undergraduate years	Who was submitted to treatment? (%)								Result of the treatment (%)							
	Myself		Family member		Close friend		Nobody		Ineffective		Not very effective		Quite effective		Don't know	
	Hom	Acu	Hom	Acu	Hom	Acu	Hom	Acu	Hom	Acu	Hom	Acu	Hom	Acu	Hom	Acu
1^st^ year	42.3	9.9	21.6	43.2	19.8	23.4	16.2	23.4	6.4	1.2	15.0	11.8	49.5	62.3	29.0	24.7
2^nd^ year	35.3	9.4	22.3	40.0	16.5	27.0	25.9	23.5	12.7	3.1	17.5	12.3	46.0	61.5	23.8	23.1
3^rd^ year	31.5	21.9	34.2	42.5	16.4	19.2	17.8	16.4	10.0	3.3	25.0	27.9	30.0	49.2	35.0	19.7
4^th^ year	37.5	29.7	31.2	31.2	17.2	31.2	14.1	7.8	18.2	5.1	23.6	20.3	30.9	61.0	27.3	13.5
5^th^ year	30.6	20.0	24.7	38.8	27.0	28.2	17.6	12.9	24.3	6.7	27.1	13.5	24.3	60.8	24.3	18.9
6th year	30.3	31.8	25.7	25.7	22.7	24.2	21.2	18.2	34.6	5.5	25.0	27.8	26.9	61.1	13.5	5.5
**% Total**	**34.6**	**20.4**	**26.6**	**36.9**	**19.9**	**25.5**	**18.8**	**17.0**	**17.7**	**4.1**	**22.2**	**18.9**	**34.6**	**59.3**	**25.5**	**17.6**

*Hom = Homeopathy; Acu = Acupuncture.*

In relation to the main indications for these therapies, 37% of all the undergraduates believed that these therapies should be indicated only for patients with chronic diseases, while 19.5% (for homeopathy) and 38.2% (for acupuncture) also believed that these therapies could also be effective in the treatment of acute diseases. Overall, 27% (homeopathy: 36.0%; acupuncture: 17.8%) did not have an opinion. Other students (homeopathy: 4.5%; acupuncture: 2.3%) added alternative responses: "no indicator", "psychosomatic diseases" and "diseases without response to other treatments". Most of the comments related acupuncture to "treatment of acute or chronic pain" (Table 4).

**Table 4:** Medical students’ perceptions of the main indications for homeopathy and acupuncture and the general effectiveness of such therapy (Faculdade de Medicina da Universidade de São Paulo; n = 484)

Undergraduate years	What are the main indications? (%)								What is its general effectiveness? (%)							
	Acute diseases		Chronic diseases		Both		Don't know		Ineffective		Not very effective		Quite effective		Don't know	
	Hom	Acu	Hom	Acu	Hom	Acu	Hom	Acu	Hom	Acu	Hom	Acu	Hom	Acu	Hom	Acu
1^st^ year	42.3	36.0	4.5	5.4	18.0	29.7	35.1	28.8	5.4	0.9	27.9	10.8	38.7	68.5	27.9	19.8
2^nd^ year	37.6	36.5	4.7	4.7	24.7	37.6	30.6	20.0	10.6	2.3	25.9	16.5	30.6	55.3	32.9	25.9
3^rd^ year	39.7	43.8	4.1	1.4	17.8	28.8	37.0	26.0	10.9	2.7	27.4	19.2	21.9	49.3	39.7	28.8
4^th^ year	28.1	29.7	1.6	6.2	21.9	45.3	37.5	10.9	12.5	4.7	31.2	15.6	21.9	70.3	34.4	9.4
5^th^ year	34.1	36.5	1.2	3.5	21.2	47.0	41.2	11.8	25.9	0	23.5	12.9	14.1	64.7	36.5	22.3
6th year	40.9	37.9	1.5	9.1	13.6	40.9	34.8	9.1	30.3	7.6	34.8	21.2	19.7	65.1	15.1	6.1
**% Total**	**37.1**	**36.7**	**2.9**	**5.0**	**19.5**	**38.2**	**36.0**	**17.8**	**15.9**	**3.0**	**28.4**	**16.0**	**24.5**	**62.2**	**31.1**	**18.7**

*Hom = Homeopathy; Acu = Acupuncture.*

Questioned about the general effectiveness of these treatments, some of the students answered that they were ineffective (homeopathy: 15.9%; acupuncture: 3.0%), while a group believed in some level of effectiveness (homeopathy: 52.9%; acupuncture: 78.2%), coming close to the figures obtained from the question concerning the effectiveness of their own treatment or that of a person they knew well. Another group (homeopathy: 31.1%; acupuncture: 18.7%) did not know how to evaluate the effectiveness, stating that "it depends on the indication", "there is a lack of research" and "it is attributable to the placebo effect" (Table 4).

Around 35% of the undergraduates were receptive towards making these therapies available for public primary care services, while 26.6% (homeopathy) and 42.4% (acupuncture) also defended the availability of these treatments in hospitals, and not only in public primary care clinics. The group that was contrary to such initiatives (homeopathy: 28.7%; acupuncture: 10.5%) presented the following justifications: "they should not be offered because there is no scientific proof for them" and "the public services don't have time for long conversations". Overall, few students had no opinion about this (homeopathy: 6.8%; acupuncture: 3.6%). (Table 5)

**Table 5:** Medical students’ opinions on whether homeopathy and acupuncture should be offered within public healthcare and integrated within conventional medical services (Faculdade de Medicina da Universidade de São Paulo; n = 484)

Undergraduate years	Should they be offered within public healthcare? (%)								Should they be integrated within conventional medical services? (%)					
	No		In hospitals		In clinics		In both clinics and hospitals		No		Yes		Don't know	
	Hom	Acu	Hom	Acu	Hom	Acu	Hom	Acu	Hom	Acu	Hom	Acu	Hom	Acu
1^st^ year	15.3	6.3	9.0	11.7	29.7	23.4	38.7	55.8	11.7	3.6	58.5	76.6	29.7	19.8
2^nd^ year	21.2	10.6	8.2	11.8	31.8	32.9	34.1	40.0	14.1	9.4	52.9	61.2	32.9	29.4
3^rd^ year	27.4	13.7	1.4	5.5	28.8	32.9	32.9	43.8	20.5	9.6	45.2	61.6	34.2	28.8
4^th^ year	25.0	7.8	3.1	4.7	37.5	39.0	20.3	40.6	21.9	9.4	48.4	73.4	29.7	17.2
5^th^ year	36.5	4.7	2.3	5.9	36.5	49.4	20.0	37.6	27.0	5.9	47.0	81.2	25.9	12.9
6th year	47.0	19.7	1.5	4.5	37.9	39.4	13.6	36.4	43.9	19.7	37.9	66.7	18.2	13.6
**% Total**	**28.7**	**10.5**	**4.2**	**7.3**	**33.7**	**36.2**	**26.6**	**42.4**	**23.2**	**9.6**	**48.3**	**70.1**	**28.4**	**20.3**

*Hom = Homeopathy; Acu = Acupuncture.*

Regarding the possibility of integration with conventional medicine in public health-care, 48.3% were in favor of this for homeopathy and 70.1% for acupuncture, while 28.4% (homeopathy) and 20.3% (acupuncture) could not decide about this. The ones who were against the idea (homeopathy: 23.2%; acupuncture: 9.6%) justified themselves by saying that "the attempt to integrate would be fruitless", and the defenders of the idea cited the benefits of "lower costs for the services (referring to medicines)" and "humanization of the medical services" (Table 5).

## DISCUSSION

The high percentage of questionnaires that were correctly filled out (94.53%) indicates a high degree of interest in this subject, considering that the participants were under no obligation to respond to the questionnaire. The prevalence of male students among the participants corresponded to their proportion within the student population of FMUSP, which has more male than female students.

The fact that the students were receptive towards including the subjects of homeopathy and acupuncture within the basic curriculum (an average of 72% in favor of this as an option, and 19% as a compulsory subject) backs up other surveys carried out in the United Kingdom (69% out of 592 students interviewed at the University of Glasgow^19^) and in Germany (an average of 49% out of 140 students interviewed at the University of Düsseldorf^20^ and 43% out of 204 students interviewed at the University of Marburg^21^). The medical undergraduates’ general interest in learning about CAM was also high in other surveys carried out in Canada (65% of the students^22^) and in the United States (43% of the students^23^ and 72% of the students^24^).

The FMUSP students’ justifications for learning about CAM within their course finds backing from other surveys.^25^ The Association of American Medical Colleges^26^ has declared that medical undergraduates should have enough knowledge about CAM for them to be able to advise their patients regarding the possible benefits and harm from each therapy during their medical practice.

Students’ level of knowledge about CAM was low in the present study, and similar to results from other surveys done among undergraduate students,^27^ resident physicians^28^ and physicians.^8^ Such low levels of knowledge contribute towards the growth of prejudice concerning these therapeutic practices within the medical sector.

Reinforcing the need for medical education regarding CAM, a questionnaire presented to undergraduates at the medical school of one of the largest teaching hospitals in the United States^29^ showed that the perception of the effectiveness of these therapies was directly related to the level of knowledge regarding this subject.

In a similar way to what was observed in our survey, a questionnaire among American physicians^8^ showed that belief in the effectiveness of CAM and willingness to recommend it to patients were strongly associated with these physicians’ own experience with such therapies.

Just as mentioned by our students in their responses to the questionnaire, the main reason given by other specialists for indicating CAM treatment to their patients, was that "the patient's disease did not react to conventional treatments".^30,31^

Supporting the responses by a majority of the students we interviewed, in which some effectiveness was attributed to homeopathy and acupuncture, a meta-analysis^5^ of twelve surveys on physicians’ conventional attitudes regarding CAM practices has shown that physicians usually consider that these therapies have moderate effectiveness.

Emphasizing the perspicacity of the answers of most of the undergraduates interviewed, which were favorable towards the integration of homeopathy and acupuncture into conventional medicine in public healthcare, initiatives developed in India^32^ have shown that service quality was increased and some improvement in the cost/benefit relationship occurred when such integration was duly implemented.

In relating some of the students’ attitudes to the academic training process, we observed that there was a little resistance in accepting these therapeutic practices among the students in the later years (5^th^ and 6^th^ years), especially in relation to homeopathy. We noted that, at this level of the medical course, there was an increased sense of criticism, governed by a demand for scientific evidence that would give a basis for the various therapeutic procedures. From a general analysis of students’ responses, our supposition is that their relative preference for acupuncture is related to the acupuncture activities (research, teaching, primary care clinics, etc.) that have been developed at FMUSP over the last few years. This will have transmitted technical and scientific backing to students, regarding the applicability of acupuncture. This contrasts with the situation for homeopathy, for which we consider that educational and research projects are only now starting to be developed.

To satisfy the demand from students, FMUSP has now included in its curricular framework two optional subjects called "Fundaments of Homeopathy (MCM0773)" and "Introduction to Acupuncture (MSP0668)", taught by specialist physicians and offered to FMUSP undergraduates in their 3^rd^ and 4^th^ years. This began in 2002.

These disciplines are made available every six months, with four contact hours of instruction per week, corresponding to a total of 60 contact hours (five class credits). These classes present the basic precepts of each specialization, the scientific evidence that supports the theoretical hypotheses and their therapeutic activities, thus satisfying the main requirements^33,34^ of medical education in CAM.

Most of the initiatives concerning CAM learning in other countries involve an average of two contact hours of instruction dedicated to each therapeutic category, within courses with a maximum of 20 hours of instruction.^13^ In contrast to this, we believe that a minimum of 60 contact hours per discipline is a prerequisite for offering future physicians the knowledge that is required for them to be able to discuss these practices and suggest them to their patients. Moreover, this quantity of hours provides students with the technique that is required for them to take part in primary care teaching clinics, which will increase their experience in the clinical application of these therapies.^35,36^

The results obtained from our research among FMUSP undergraduates were similar to those from other surveys done among medical students in other countries and academic institutions.^28–36^ The majority of our medical students were interested in learning the principles of homeopathy and acupuncture, and were receptive to including these disciplines within the undergraduate curriculum. Even with little previous knowledge, they were able to observe and report on the effectiveness of their own treatment and that of people they knew well, and they valued the use of these therapies in cases of chronic disease and also for acute diseases. Although a significant proportion of the students did not know the main reasons for using these unconventional therapies, or how effective they may be, most of the students defended the use of these medical specializations in public healthcare. They envisaged the integration of these therapies into conventional medicine, thereby seeking to increase the physician-patient relationship and enlarge the spectrum of action of the doctor's skills.

## CONCLUSIONS

The medical students were interested in learning the principles of homeopathy and acu-puncture, were able to observe and report on the effectiveness of these treatments and defended the use of these medical specializations within public healthcare. Expansion of this initiative for teaching homeopathy and acupuncture as elective disciplines within the undergraduate course will hopefully, in the future, take this teaching activity to master's degree level and to medical residence within FMUSP. This will allow an academic approach to be adopted and will encourage increased clinical quality and scientific research regarding these therapies.
